# Virtual practice facilitation as an implementation strategy for launching opioid safety committees for quality improvement in primary care: feasibility, acceptability, and intervention fidelity

**DOI:** 10.1186/s12875-024-02632-w

**Published:** 2024-10-26

**Authors:** Jessica Mogk, Claire L. Allen, Carly E. Levitz, Kelsey Stefanik-Guizlo, Emily Bourcier, Melissa Trapp Petty, Paula Lozano

**Affiliations:** Kaiser Permanente Washington Health Research Institute, 1730 Minor Ave, Ste 1600, Seattle, WA 98101 USA

**Keywords:** Practice facilitation, Implementation facilitation, Practice coaching, Virtual practice facilitation, Quality improvement, Opioid safety, Interdisciplinary chart review, Pain management, Persistent pain

## Abstract

**Background:**

Practice facilitation (PF) is an evidence-based multicomponent in-person implementation strategy. COVID-19-related lockdowns caused many implementation initiatives to rapidly shift to virtual settings, but there is limited evidence on PF deployed exclusively using virtual meeting platforms. Our objective was to assess the feasibility and acceptability of virtual PF used in a primary care setting to implement interdisciplinary opioid safety committees (OSCs) to improve care for patients using opioid medicines for persistent pain and reduce high-dose opioid prescribing. We also describe alignment of virtual PF with the core functions of PF and fidelity of participating clinics to the OSC intervention.

**Methods:**

We applied qualitative and quantitative methods to evaluate virtual PF used to implement a quality improvement project at Kaiser Permanente Washington, an integrated health system in Washington State. We established interdisciplinary OSCs in primary care clinics using virtual PF. OSCs were tasked with promoting opioid safety and high-quality pain care through population management and chart reviews. We used administrative data to calculate feasibility measures including attendance and retention. Acceptability data came from interviews with OSC members conducted by evaluators. Measures of fidelity to the OSC intervention were abstracted from meeting notes and administrative data. We used qualitative methods to assess the adherence of virtual PF to the core functions of PF.

**Results:**

Facilitators carried out a comprehensive PF approach virtually and demonstrated adherence to the core functions of PF. We established OSCs in eight clinics and conducted an average of 17.5 virtual PF meetings over eight months of PF for each clinic. Average attendance was 75% and we had 84% retention. OSC members were highly satisfied with virtual PF. Facilitators effectively supported teams through implementation and technical challenges and OSC members gained skills through virtual PF. We implemented OSCs with high fidelity, suggesting virtual PF is an effective implementation strategy.

**Conclusions:**

We found virtual PF is a feasible and acceptable implementation strategy for this intervention and identified strategies to support care teams through challenges. Our findings can help inform future implementation efforts, especially those hoping to engage geographically dispersed clinics or remote clinical staff.

**Trial registration:**

Not applicable.

**Supplementary Information:**

The online version contains supplementary material available at 10.1186/s12875-024-02632-w.

## Background

Practice facilitation (PF) is well-established as an effective multicomponent in-person implementation strategy [1, 2]. PF has been used in healthcare to support the adoption of evidence-based practices, build quality improvement (QI) capacity, and improve clinical outcomes [3–5]. Taylor and colleagues define the core functions of PF as (1) helping clinics organize, prioritize, and sequence QI activities, (2) training clinic staff to understand and use data to drive QI, (3) increasing clinic capacity for QI activities, (4) helping build a team orientation among clinic staff and a clinic culture receptive to change, and (5) sharing best practices and lessons across clinics [6].

The COVID-19 pandemic created widespread challenges for in-person meetings that are part of the traditional PF approach [7] and forced many implementation initiatives to shift to virtual settings [8–12]. While PF has been deployed with a mix of in-person and virtual meetings [5, 13–16] and with in-person meetings and virtual support between meetings [17], there are few studies describing the use of PF in an exclusively virtual mode.

We define virtual PF as PF led remotely by a practice facilitator using an online meeting platform such as Zoom or Microsoft Teams. During virtual PF, facilitators may lead meetings with voice only or employ tools such as video conferencing and screen sharing. Care team members engaged in virtual PF may join meetings by phone, on individual laptops/computers, or as a group on a single device. Some studies report using virtual PF without describing it in detail [18–20], or describe a form of virtual facilitator support that is less comprehensive than traditional PF [21]. Many studies do not specify whether their facilitation was virtual or in-person [22–26]. Behling and colleagues used a comprehensive virtual PF approach, but their manuscript is focused on the program’s outcomes rather than virtual PF as a strategy [27]. Recent efforts have illuminated best practices and strategies for virtual PF [28, 29] but there is little knowledge about the adherence of virtual PF to the core functions of PF or the feasibility and acceptability of virtual PF [30]. This manuscript was written to share our experience with virtual PF to help project teams make more informed decisions about whether to conduct PF in-person or virtually.

PF has been successfully employed in primary care as an implementation strategy to improve the quality and guideline adherence of care for patients with long-term, persistent pain, including reducing high-dose opioid prescribing [31, 32]. We used virtual PF to implement interdisciplinary Opioid Safety Committees (OSCs) at Kaiser Permanente Washington, an integrated health system in Washington State. OSC implementation was one part of a larger QI initiative to reduce high-dose opioid prescribing and improve care for patients with pain. Outcomes of the larger initiative will be reported elsewhere. The OSC intervention was based on the Six Building Blocks for improving opioid management and informed by the Chronic Care Model [32–36].

The objective of this evaluation was to assess the feasibility of virtual PF as the primary implementation strategy for establishing interdisciplinary OSCs to reduce high-dose opioid prescribing in primary care clinics and the acceptability of virtual PF among primary care teams. We also examine the fidelity of participating clinics to the OSC intervention. Additionally, we describe examples of the core functions of PF delivered virtually, lessons learned, and recommend strategies for addressing challenges with virtual PF.

## Methods

### Context and intervention

This work was part of a QI project led by researchers at the Center for Accelerating Care Transformation (ACT Center) [37] at Kaiser Permanente Washington Health Research Institute (KPWHRI). The ACT Center provides scientific support for health care delivery initiatives as part of our Learning Health System partnership with Kaiser Permanente Washington [38]. The KPWHRI Institutional Review Board determined this project was not human subjects research. We used the Standards for Reporting Implementation Studies checklist [39] (Supplement A) and the Consolidated Criteria for Reporting Qualitative Research checklist [40] (Supplement B) to report our methods and findings.

Virtual PF was the primary implementation strategy for establishing OSCs to improve opioid safety and pain care in primary care clinics. The OSC model was initially codesigned with one primary care clinic using in-person PF from August 2019 to March 2020. COVID-19 and the resulting restrictions on in-person meetings caused us to shift our design and implementation work to a virtual format.

OSCs are interdisciplinary teams with protected meeting time to review patient charts and provide care recommendations to promote opioid safety and quality pain care. We defined an OSC as including at least one member from each of these five roles: primary care provider (PCP), pharmacist, social worker, clinic leader, and one additional care team member (e.g., nurse or medical assistant). Intervention milestones were (1) launching the OSC with a kickoff meeting, (2) assessing the current state of care for patients with persistent pain, (3) setting QI priorities, (4) establishing a chart review process, (5) completing at least one plan-do-study-act (PDSA) cycle on a priority topic, (6) creating an OSC charter, and (7) establishing a follow-up process for patients previously reviewed by the OSC. For meetings during which the OSC reviewed patient charts, the patient’s PCP was encouraged to attend. Sometimes the patient’s PCP was a standing OSC member, but often the patient’s PCP was an additional provider from the clinic who joined the OSC meeting as a guest. Participation of the patient’s PCP in chart reviews was considered a core piece of the intervention because the codesign clinic found that the patient’s PCP contributed important context to the review and recommendations from OSC reviews could be more easily carried out if the PCP was involved in the process.

We used virtual PF to establish OSCs at eight primary care clinics between June 24, 2020, and June 30, 2023, designated as Clinics A through H. Some intervention design work was still underway during implementation at Clinics A and B. Clinic G had an established non-interdisciplinary chart review processes prior to this initiative. Clinics were selected in partnership with care delivery leaders based on below-target performance on opioid prescribing measures and leadership support for launching an OSC. Implementation timing was driven by leadership prioritization, clinic capacity, and PF availability. Implementation was paused intermittently due to competing priorities for overburdened care teams throughout the COVID-19 pandemic.

Each clinic received seven to nine months of virtual PF support from Master’s-level researchers trained in QI who dedicated about four hours per week to each clinic during active PF. One facilitator (JM) led work with six clinics, and an additional facilitator (KSG) led work with two clinics.

Virtual PF was conducted using Microsoft Teams because all Kaiser Permanente Washington employees had Teams on their computers. Except for the kickoff meeting for Clinic C (which was in-person), facilitators joined all meetings virtually using video and voice. Care team members typically joined PF meetings from individual laptops or by phone and occasionally joined as a group on a single device in a meeting room. Facilitators engaged teams between meetings through email, Teams chats, and one-on-one virtual meetings. Meeting materials were made available to OSCs on Microsoft Teams.

### Evaluation design and outcomes

We used qualitative and quantitative methods to evaluate virtual PF’s feasibility and acceptability, as defined by Proctor and colleagues (Table 1) [41]. We also evaluated fidelity to the OSC intervention as a measure of the effectiveness of virtual PF [42]. We used qualitative methods to evaluate the adherence of virtual PF to the core functions of PF [6].

**Table 1:** Implementation outcomes, definitions, data sources, and measures

Outcome	Definition	Data source	Measures
Feasibility of virtual PF	Extent to which virtual PF can be successfully carried out in a given setting	Administrative data	Virtual PF meetings held (total #) Virtual PF meetings held as planned (%) Attendance Retention
Acceptability of virtual PF	Perception that virtual PF is agreeable, palatable, and/or satisfactory	Qualitative interviews	Feedback from care teams
Fidelity to the OSC intervention	Degree to which OSCs were implemented as intended; assessment of the potential effects of virtual PF as an implementation strategy[42]	Administrative data	Interdisciplinary representation Milestone completion Chart reviews completed as planned Patient’s PCP present for review

### Data collection and analysis

Facilitators recorded administrative data in detailed meeting notes in Microsoft OneNote and by entering structured data describing their activities in Microsoft Forms after each PF meeting. Administrative data were used to calculate feasibility measures including number of meetings, attendance, and retention. Measures of fidelity to the OSC intervention were also abstracted from administrative data, including PF support activities, OSC membership, milestone completion, chart review completion, and patient’s PCP attendance at OSC meetings. Meeting notes were used to add context to feasibility and intervention fidelity measures, such as the reasons meetings or chart reviews did not occur as planned and barriers to attendance, retention, and engagement in virtual PF.

Evaluators separate from the implementation team (CL, EB, MTP) led the qualitative data collection and analysis. Evaluators had graduate degrees in public health, health administration, and epidemiology, and they each had 5–20 years of qualitative analysis experience. Qualitative data was collected using pragmatic approaches. To recruit OSC members to participate in interviews, facilitators started an email thread with the OSC members to introduce the evaluators, and evaluators followed up with information about the evaluation and an invitation to participate in interviews. Interviews were conducted in virtual meetings using an interview guide (Supplement C) which invited OSC members to share about successes, challenges, implementation strategies, and feedback regarding their experience with virtual PF and the OSC intervention. Interviews were typically one-on-one and lasted 25 min on average. Interviews were recorded and transcribed using Microsoft Teams. Participants received recognition in an employee rewards platform used by Kaiser Permanente Washington. For participants who were not PCPs, this recognition included points that could be used for online purchases worth about $45 (PCPs were not eligible for points in this platform).

Evaluators analyzed OSC member interview transcripts using thematic analysis methods [43]. First, transcripts were coded by a single evaluator in ATLAS.ti [44] using a high-level code list which was based on the interview guide and designed to abstract data regarding OSC member experience, successes and challenges, and lessons learned. Coded data was exported into an Excel spreadsheet, which evaluators reviewed iteratively as a team to ensure consistent use of codes and inductively identify themes within the high-level codes, first across interviewees and then across clinics. Data was subsequently synthesized into code memos with themes and quotes labelled by clinic. The evaluators and the implementation team reviewed the memos at multiple points during the QI project to inform implementation and evaluate the resonance of themes. While evaluators collected and analyzed data regarding many aspects of the QI project, only data related to OSC member’s experiences with virtual PF are presented in this manuscript.

## Results

### Delivery of the core functions of PF using virtual PF

The facilitators carried out a comprehensive PF approach that spanned the five core functions of PF [6] in a virtual setting. Table 2 provides examples of PF activities delivered in all clinics using virtual meetings. We also provide quotes from care team members about their experiences with core functions of PF in this project to add depth to the examples.

**Table 2:** Core functions of PF delivered virtually

Core function of PF[6]	Examples of core functions of PF delivered virtually	Quotes from OSC members regarding core functions of PF carried out virtually
Help the practice organize, prioritize, and sequence QI activities	• Facilitating clinic self-assessments • Leading a prioritization activity to focus teams’ work	*“Giving us a goal*,*helping us determine our goal by being able to clarify things we were talking around and around. It was helpful. We all wanted the same thing but sometimes weren’t using the right words.”* (Clinic A)
Train practice staff to understand and use data to drive QI	• Sharing measures over time using a data visualization software • Helping teams understand empanelment and electronic data capture • Using data for chart selection	*“[The facilitator] came back and provided concrete data on how we’ve been doing in terms of our improvement of opioid amounts in the clinic*,*or new patients started on opioids*,*naloxone prescriptions. She did it by provider and showed a graph on how we’re doing. That was so helpful… From the data*,*we can see what we need to work on*,*where we need to target*,*and then we can re-evaluate that and see if we’ve made improvements.”* (Clinic B)
Increase practice capacity for QI activities	• Working through PDSA cycles (for example, to test different ways to share educational resources with patients or creating a new shortcut in the electronic health record) • Using chart review as an opportunity to identify and address system issues • Completing follow-up monitoring for patients already reviewed	*“Doing the PDSAs and understanding how those work has been helpful. Helps things go a little smoother and see those end goals …and that it’s okay to make changes in between…PDSA keeps things on track and keeps people accountable -it gives everyone a role to play.”* (Clinic D)
Help build a team orientation among practice staff and a practice culture receptive to change	• Establishing an interdisciplinary OSC and patient review process • Identifying ways other care team members could support PCPs in caring for patients with pain	“*[The facilitator] did make sure that everyone had their voice. She was fair and she was really good at helping us collaborate.”* (Clinic A)
Share best practices and lessons across practices	• Sharing tools and updates across clinics • Sharing ideas and tips across clinics • Building connections with quality leaders	*“She would give us helpful hints that were helpful for other clinics or if she saw us trying something that hadn’t been successful*,*she would say go ahead and give it a try. Let me know if it works out for you. She just did a really great job of putting it together.”* (Clinic F)

### Feasibility of virtual PF for primary care teams

Table 3 displays measures of feasibility of virtual PF for primary care teams. Facilitators conducted an average of 17.5 meetings at each clinic during the active PF period (7–9 months). The duration of PF and the number of meetings was intentionally reduced after implementation in Clinics A and B to improve efficiency. 94.0% of all meetings were held as planned (range 81.3–100% across clinics). Most planned meetings that did not occur were cancelled last-minute, either because OSC members were called away for patient care or because the pre-work for an OSC review had not been completed. Attendance of required team members ranged from 67.7 to 81.1% across clinics, with an average of 75.1%. Attendance by provider type (i.e., PCP, pharmacist, social worker, clinic leader, and additional care team member) ranged from 69.8% (social worker) to 77.9% (primary care provider). Attendance data was missing from 14.0% of meetings (mostly from Clinics A and B, who had meetings before administrative tracking processes were finalized); these meetings were excluded from the attendance measure. Common barriers to attending meetings which were documented in meeting notes included difficulty protecting meeting times (i.e., blocking off time that would otherwise be used for patient care), other scheduling conflicts, and getting pulled away to deal with urgent clinic issues. Of the 63 individuals involved in the OSCs, 53 attended meetings throughout implementation (84.1% retention). OSC members who stopped attending meetings did so because they changed roles or left the organization (*n* = 5), did not think their attendance was necessary (*n* = 4), or did not have the capacity to participate (*n* = 1).

**Table 3:** Feasibility of virtual PF

Measure	Clinic A	Clinic B	Clinic C	Clinic D	Clinic E	Clinic F	Clinic G	Clinic H	Average
# of virtual PF meetings held	25	27	17	13	15	13	16	14	17.5
% of virtual PF meetings held as planned	96.2	96.4	94.4	81.3	100	100	94.1	87.5	94.0
Average % of required attendees present at virtual PF meetings*	77.6	68.7	72.5	77.8	74.4	80.7	67.7	81.1	75.1

*Attendance data is missing for 20 PF meetings (missing 7 from Clinic A, 8 from Clinic B, 1 from Clinics C and D, 2 from Clinic F, and 1 from Clinic G). These meetings were excluded from this measure

Meeting notes revealed that virtual PF created some challenges for care teams, especially when care team members had low technology literacy. For example, while all OSC members had access to Teams prior to virtual PF, some OSC members reported low familiarity with the application, especially document storage and sharing functions. For most clinics, facilitators demonstrated how to document the chart review in Teams and share the review with the patient’s PCP multiple times before these tasks were taken on by OSC members. Facilitators also had difficulty communicating with care team members between meetings (e.g., reaching them via email or Teams chat). This may have been due to technical difficulties or competing priorities for care team members.

Unequal access to, and engagement with, technology to support virtual meetings also emerged as a theme in meeting notes. While many OSC members had individual laptops with cameras and microphones, members without adequate equipment participated exclusively by phone, gave input by chat only, or joined meetings with another team member on their laptop. When more than two OSC members joined on the same device (i.e., hybrid participation), it was difficult for the facilitator to hear and engage everyone in the room. In addition, members with cameras often chose not to use them.

One PCP on an OSC reported dissatisfaction with using Teams for meetings and document storage, and transitioned OSC activities to in-person after virtual PF had ended. The seven other OSCs continued using Teams for OSC meetings and documentation.

### Acceptability of virtual PF for primary care teams

Interviews were conducted with 47 OSC members (out of 53 invited, 88.7% response rate), with a range of 4 to 7 per clinic. Themes from the qualitative interviews related to virtual PF were: (1) Positive feedback about the facilitators, (2) Tools used for virtual PF were useful, (3) Facilitators were supportive and resourceful, (4) Facilitators were organized, responsible and reliable, (5) Facilitators fostered trust, (6) Virtual PF helped teams work through challenges, and (7) OSC members gained skills through virtual PF. Exemplar quotes for each of these themes are presented in Table 4. In this section, we highlight qualitative findings related to the acceptability of virtual PF in our context.

**Table 4:** Qualitative themes related to virtual PF

Theme	Exemplar quote(s)
Positive feedback about the facilitators	*“[Name] was our facilitator- she was great; she let us know it was our OSC and our plan-she was just there to help. She was the perfect facilitator. She was good at walking the line of doing and encouraging people to take ownership.”* (Clinic H) *“I really liked [our facilitator]. She did a very good job as far as teaching us what we needed to do and how to get it done. And making sure we had ideas. She was very willing to listen to new ideas and we made some scripting changes – sometimes we talked about things like that. She kept us on track.”* (Clinic H) “*All of the practice facilitators were very*,*very cheerful*,*eager to help make improvements. It brought a nice feeling to it. They brightened the mood and were really motivated to improve.”* (Clinic A)
Tools used for virtual PF were useful	*“I think having all of that like essentially given to us in terms of like the template and layout [in Teams] … those are just not intuitive things for me… So I think that was really helpful.”* (Clinic G)
Facilitators were supportive and resourceful	*“I think [the facilitator] was very supportive. She was very good about sending emails*,*messages and doing the audits sheets*,*taking and answering questions-not one thing specific-she was there if we needed anything. If she couldn’t do it*,*she would find someone who could.”* (Clinic H) *“She was very understanding and didn’t make anyone feel bad for anything. Like if there’s a case that was challenging or some clean-up to do with medication management*,*there was no judgement or any negative feedback. It was all supportive and for the case of making it better for everyone*,*patient included.”* (Clinic G)
Facilitators were organized, responsible, and reliable	*“I appreciated [our facilitator’s] organizational skills. She really helped to get us to understand what our mission was*,*what the purpose of the committee was*,*and how we could help the providers.”* (Clinic F) “*I would say [the facilitator was] very organized. I remember after all of the meetings*,*there was a summary of what the meeting was*,*what the action items were*,*and that helped with follow-through for things like this that aren’t on my daily to-do list*,*to have that available to me at the end of the meeting*,*so I could meet the next deadline. That was the most helpful thing and the thing that stands out the most.”* (Clinic B) *“Something seemingly simple*,*like the act of renaming the document made a world of difference. [The facilitator] did a great job at helping us stay organized in a lot of instances. If that hadn’t happened*,*we would have had difficulty staying on task or even up to date with some of those. That was a huge help.”* (Clinic A) *“I would say [the facilitator was] very eager*,*very positive*,*super helpful. I think incredibly patient. Sometimes it would be a while before at least myself would respond to emails – so they were making sure that things were followed up on. They were also very organized.”* (Clinic B)
Facilitators fostered trust	*“In the first meeting I thought I don’t know who this [facilitator] is*,*but she had her agenda set for what to talk about and she kept the flow going really well. She helped it go very seamlessly and helped keep everybody on track and everybody’s components have equal footing. So*,*it wasn’t all about social work or the providers opinion on patient – it was truly keeping it integrated- to look at all aspects of it not just taking a narrow view of the problem like it was the provider’s fault.”* (Clinic E) *“[The facilitators] asked for opinions based on what your specialty is – they were always asking if there was something missing from each perspective*,*trying to get all around care required to make sure nothing is missing; when I was brand new I didn’t know any of the terminology or acronyms- they were helpful answering questions*,*not at all making me feel like an idiot because I didn’t know.”* (Clinic G)
Virtual PF helped teams work through challenges	*“[The facilitator is] very thorough. She’s engaged*,*not just there to facilitate but make sure we’re on track and on time. She’s been great – really easy going. Cause some of us get stuck in a rut or we’re not sure – oh we tried looking for info and couldn’t find it – she’ll have ideas like have you tried that?”* (Clinic D) *“The thing I appreciated was she helped us get over…helped describe what we’re doing*,*why we’re doing it*,*kept us on track*,*made sure we continued to meet and review. I think she was very helpful in facilitating the conversation and helping everyone get involved. When we run [the OSC meetings] now everybody understands their role. That’s what a facilitator should do is get us going then let us fly and [our facilitator] was great with that.”* (Clinic G)
OSC members gained skills through virtual PF	*“I learn a lot from the people that are on the committees. They have different levels of expertise themselves. They come from different perspectives*,*which helps me gain new [perspectives]-- pharmacy has a specific way of looking at things – but that’s not the only way to look at it. Learning from other people their perceptions of these issues is valuable to me and helps me understand how to move forward with these situations.”* (Clinic E) *“Working with [the facilitator on this project] has really opened my eyes a lot more to my role in management of people with chronic pain and people taking opioids long term.”* (Clinic C)

Across all clinics, OSC members were highly satisfied with virtual PF. They shared positive comments about virtual PF tools and the approach taken by facilitators.

One OSC member shared:

*“I think we were just really set up for success the way that [the facilitator] has the program set up. She has a ton of resources that*,*if we forget something as we’re kind of moving through it*,*that we can access. Everything is kind of step-by-step. She sent us tutorials and stuff at first so we could get our feet wet. She’s really just made it user-friendly.”* (Clinic F).

OSC members described facilitators as approachable, supportive, and reliable. One OSC member said: “*[Even though] we only see her face every couple of weeks*,*she was totally available”* (Clinic D). OSC members also shared how facilitators built trust in a virtual environment:

*“Trust has been fostered through empowering the team to take ownership of it. [The facilitator] has been cheerleading us on. Initially giving us information on the forms and then just being there to support us through the process and validating the steps we’ve been taking.”* (Clinic C).

Facilitators helped teams work through challenges and focus on patient-centered improvements:

*“The Committee wasn’t that motivated to start with. [The facilitator]*,*through her perseverance*,*got us involved. She got us to be active participants and then she bowed out. It’s a tough position she was in to get people motivated. But she was persistent*,*she didn’t give up*,*she seemed to believe in the process so other people bought in and she bowed out appropriately.”* (Clinic D).

*“[Our facilitator] was great. She actually held the line on us because there were some flippant comments and she called us out on them which was good. She said these patients are in pain and we don’t get to make snarky comments.”* (Clinic E).

Finally, OSC members gained concrete skills by engaging with virtual PF:

*“I learned Teams a lot more! How to navigate it. [The facilitators] were running all our forms…then slowly each week they would back off of one thing and have us do it. Obviously*,*we can’t have [the facilitators] forever. I learned a lot of maneuvering in Teams I wouldn’t have got otherwise…”* (Clinic C).

*“[The facilitator] helped me understand [opioid use] from a medical point of view*,*and the pressure on the physicians and how they taper. I understand that whole process more. My role is to support the patient and support the physician as well.”* (Clinic A).

No OSC members identified challenges in the partnership with the facilitator, even though interviewees were asked directly about this.

### Fidelity to the OSC intervention

The goal of virtual PF in this project was to implement OSCs in eight clinics. Administrative data shows that this was accomplished with high fidelity (Table 5). All clinics successfully established OSC teams including the recommended five roles represented to meet our definition of interdisciplinary. OSC team composition varied by clinic, and many had more members than the recommended minimum (OSCs had 7.9 members on average).

**Table 5:** Measures of fidelity to the OSC intervention

Measure	Clinic A	Clinic B	Clinic C	Clinic D	Clinic E	Clinic F	Clinic G	Clinic H	Average
Number of required interdisciplinary roles represented on OSC (5 total)*	5	5	5	5	5	5	5	5	100
Milestones completed (7 total)†	7	7	7	7	7	7	6	7	98.2
% of virtual PF meetings* where chart reviews were completed as planned	100	100	100	90.0	81.8	77.8	100	91.7	93.3
% of virtual PF meetings‡ where the patient’s PCP was present for the review	100	66.7	88.9	62.5	66.7	100	26.7	100	70.6

*Recommended interdisciplinary roles: primary care provider (PCP), pharmacist, social worker, clinic leader, and one additional care team member (such as a nurse or medical assistant)

†Intervention milestones were (1) launching the OSC with a kickoff meeting, (2) assessing the current state of care for patients with pain, (3) setting QI priorities, (4) establishing a chart review process, (5) completing at least one plan-do-study-act (PDSA) cycle on a priority topic, (6) creating an OSC charter, and (7) establishing a follow-up process for patients previously reviewed by the OSC

‡Of meetings that occurred (i.e., excluding meetings that were cancelled)

Virtual PF meetings were designed to help the OSC teams accomplish the intervention milestones including establishing a chart review process. Virtual PF meetings which were not used for chart review were focused on the other milestones. All but one clinic reached all seven milestones (Clinic G did not create an OSC charter).

Some virtual PF meetings were dedicated to completing OCS chart reviews. Across all clinics, OSCs successfully completed chart reviews at 93.3% of meetings where a chart review was planned and completed 104 chart reviews total. The most common reason for not completing a planned chart review was that the patient’s PCP did not attend the meeting (e.g., because the purpose of the meeting was not clearly communicated to them, or a clinic visit was scheduled during the meeting time). Some OSCs chose to proceed with the review without the patient’s PCP, so the PCP was present for 70.6% of the chart reviews. OSCs continued to review patient charts after virtual PF ended, suggesting intervention sustainability.

## Discussion

We found virtual PF feasible and acceptable in a primary care setting to implement OSCs to reduce high-dose opioid prescribing and improve population-based care for people with persistent pain. We successfully carried out the core functions of PF virtually and found virtual PF to be effective in implementing the intervention with high fidelity.

These findings have important implications for implementation and QI efforts that rely on PF. Other researchers have highlighted challenges with engaging remote or geographically dispersed clinics in QI work. Sutton and colleagues found facilitators spent an average of 2.2 h per meeting driving to clinics [45]. McDonnel and colleagues reported relying on many facilitators living in different locations to overcome barriers of geographic dispersion [46]. Virtual PF could be a more time- and cost-effective way to support rural clinics. Future research should investigate the differences in cost between conventional and virtual PF.

Few others have evaluated virtual PF using implementation science outcomes. Bhat and colleagues conducted a mixed-methods analysis of “longitudinal remote coaching” to implement team-based chart review [30]. Bhat and colleagues reported 58% of sessions were held as planned and attendance was 81% on average (compared to 94% and 75% in our project, respectively). Our higher success with meeting occurrence and lower attendance may be due to differences in team size. Bhat and colleagues expected only two attendees per meeting, and their absence would cause the meeting to be cancelled. In contrast, our virtual meetings could still occur even if two or more participants could not attend.

We identified several challenges with virtual PF, including low technology literacy, difficulty with online collaboration and documentation, challenges reaching care team members between meetings (e.g., via email or chat), and difficulty facilitating hybrid meetings (i.e., virtual meetings where some participants are joining together from the same room or device). To overcome these challenges, we used creative facilitation strategies including supporting team members with low technology literacy using job aids, demonstrations, and individualized support; organizing documents and team communication in a shared virtual space; using virtual channels to simulate “hallway conversations;” and optimizing technology and communication for hybrid meetings [8–12]. Hartmann and colleagues hypothesize that virtual PF is becoming more common [29]. Sutton and colleagues have observed improvements in care team technologic skills during the COVID-19 pandemic due to increased use of virtual platforms [45]. It is possible virtual PF will become more accessible with more user-friendly video conferencing tools and improved technology literacy in the healthcare workforce.

Our work has several limitations. First, our findings have limited generalizability beyond our specific context and intervention. Working in a single integrated health system where technology access was standardized aided our ability to conduct virtual PF, although we still observed variation in OSC members’ comfort using Teams. The intervention itself, which was staff-focused and could be carried out virtually, may have been easier to implement using virtual PF than an intervention directed at patients or overhauling clinic workflows. In addition, some measures, like attendance, could reflect feasibility of the intervention or feasibility of virtual PF; we cannot disentangle these. We also did not set a priori thresholds for success on feasibility measures, but our average percentage of meetings held as planned and attendance met or exceeded thresholds used by feasibility studies in the literature [30, 47, 48]. Finally, since our work occurred during and after the COVID-19 pandemic, when many activities moved to virtual settings, people may have been more receptive to virtual meetings than they would have been pre-pandemic.

## Conclusions

Our experience using virtual PF for this quality improvement project suggests PF can be conducted well virtually and that there are clear strategies to support care teams through the technical challenges of remote engagement. Our findings can help inform future quality improvement efforts, especially those hoping to engage geographically dispersed clinics or remote clinical staff. Future research is needed to compare the effectiveness and cost of different modalities of PF and to identify evidence-based best practices for virtual PF.

## Electronic supplementary material

Below is the link to the electronic supplementary material.

Supplementary Material 1

Supplementary Material 2

Supplementary Material 3

## Data Availability

The data are available from the authors upon reasonable request.

## Abbreviations

PF: Practice Facilitation
QI: Quality Improvement
OSC: Opioid Safety Committee
ACT Center: The Center for Accelerating Care Transformation
KPWHRI: Kaiser Permanente Washington Health Research Institute
PCP: Primary Care Provider
PDSA: Plan-Do-Study-Act
