# Supplementary material for: Virtual practice facilitation as an implementation strategy for launching opioid safety committees for quality improvement in primary care: feasibility, acceptability, and intervention fidelity
Source: BMC Prim Care. 2024 Oct 26;25:384. doi: 10.1186/s12875-024-02632-w (PMC11515251; doi:10.1186/s12875-024-02632-w)
Supplement: Supplementary file 3 — Supplementary Material 3 [file 12875_2024_2632_MOESM3_ESM.docx]

Opioid Safety Committee Interview Protocol

| **For Evaluation Team Only:**  Notes in brackets or italics shouldn’t be read to the interviewee. Probes that are listed as “if needed” only need to be asked if the question from which it stems doesn’t make sense to the interviewee or they need examples. Otherwise, the probes should be asked. There is suggested timing for each section.  **Goals:**   - Capture lessons about implementation strategies - Describe the experience of OSC members   **Scope: all OSC members**   - Note: The make-up of the OSCs differs among clinics.   **Interview request:** Month after active PF ends: 30 minutes, via Teams. Brief reflection prompts will be sent in advance but no expectation they prepare. |
| --- |

Introduction when the interviewee gets on Teams with you:

We are part of the Center for Accelerating Care Transformation evaluation team based at the Research Institute. The evaluation is designed to provide ongoing feedback to inform implementation and understand the impact of the Integrated Pain Management model on staff experience. This interview is for the program the practice facilitaors [include names as needed] have been working on with you. Our goal is to understand the experience of care teams in clinics implementing Integrated Pain Management, especially related to your role as a member of the Opioid Safety Committee (OSC) at [name of clinic or district].

These conversations are confidential– individual responses will not be shared beyond the evaluation team. We are distinct from the practice facilitators and neutral; this interview won’t affect your job. We will look across interviews for themes within each OSC and themes across OSCs in other clinics and districts. We will then elevate themes to share with the Center for Accelerating Care Transformation and KPWA leaders. The lessons you’ve learned will help the practice facilitators continue to improve their support and help the ACT Center to describe the implementation and impact of the OSCs.

**[Get permission to record]**

**Intro (5 min)**

1. On the OSC, what do you think you bring to the committee that other OSC members do not? This could include tools, approach, perspective, or education.
   1. *Probe if needed:* What do you see as your role on the OSC?
2. How well prepared do you feel to fulfill your role on the OSC?
   1. *Probe if needed:* What would make it easier for you to participate in the OSC?

**OSC: Most proud of, success factors, challenges (5 min)**

1. Over the past XX months [refer to implementation timeline], what OSC accomplishments are you most proud of?
2. What, if any, new knowledge or skills have you gained through participation in the OSC?
3. What have been the facilitators of making the OSC successful?
4. What have been the OSC’s biggest challenges?
   1. *Probe if needed:* What barriers have you encountered that surprised you?

**Perceptions of Integrated Pain Management implementation strategy barriers and facilitators: (9 min)**

The practice facilitators were supporting your OSC for a number of months. I’d like to hear about how your practice facilitator partnered with you and the other OSC members.

1. What qualities or behaviors in the relationship were most supportive?
   1. *Probe if needed:* What did the practice facilitator do that was helpful to you and the OSC? Did anything particular foster trust, accountability, and ownership?
2. Were there any challenges or sticky issues in the partnership between the OSC & the practice facilitators? (*If Yes:* How were these addressed?)
3. What examples come to mind of when the practice facilitator helped you improve a patient-facing care process or an OSC process?
4. Were there any challenges accessing or using the tools, forms, or information that the practice facilitators provided?

**For those involved in multiple OSCs (6 min)**

1. Which OSCs have you been involved in or are you currently are involved in? [Most pharmacists are involved in multiple OSCs]. *(if more than one, continue here; otherwise, skip to Confidence in work)*
2. How would you describe the differences between the OSCs you have been involved with?
   1. Probe: We’re interested in learning more about how OSCs can or do work together. From a KPWA perspective, what are the advantages and disadvantages of having different models of OSCs operating at the same time?
   2. Probe *but only if you have more than 5 minutes remaining in the interview*: What do you think different OSCs can learn from each other?

**Confidence in work (3 min)**

1. On a scale of 1 to 5, with 1 meaning “not at all confident” and 5 meaning “very confident,” how confident are you in your ability to keep the work of the OSC moving forward?
   1. *Probe if not already stated*: What makes you confident?
2. What support do you want more of for your OSC to help you keep the work moving?
   1. *Probe if needed:* this could be from the Medication Safety Leads or KPWA broadly

**Conclusions (2 min)**

1. What do you want me to know that we haven’t talked about?
   1. Probe: Is there anything you would like the practice facilitators or the ACT Center to know?
